# Evaluating the Quality and Readability of Online Health Information on Snapping Hip Syndrome: A Cross-Sectional Analysis

**DOI:** 10.7759/cureus.79531

**Published:** 2025-02-23

**Authors:** Mehmet Fatih Uzun, Alper Özer, Aydogan Askin, Mehmet O Atahan, Göker Yurdakul, Fatih Gölgelioğlu

**Affiliations:** 1 Orthopaedics and Traumatology, Ceylanpınar State Hospital, Şanlıurfa, TUR; 2 Orthopedics and Traumatology, Kayseri City Hospital, Kayseri, TUR; 3 Orthopaedics and Trauma, Antalya Training and Research Hospital, Antalya, TUR; 4 Orthopaedics and Traumatology, Afyonkarahisar State Hospital, Afyonkarahisar, TUR; 5 Orthopaedics and Traumatology, Yozgat Bozok University, Yozgat, TUR

**Keywords:** discern, jama benchmark criteria, online health information, readability, snapping hip syndrome

## Abstract

Aim: This study aims to evaluate the quality and readability of online health information related to snapping hip syndrome (SHS).

Methods: A cross-sectional analysis was conducted by searching the term “Snapping Hip Syndrome” on Google, Bing, and Yahoo. The first 30 results from each search engine were assessed, and duplicate or irrelevant websites were excluded. The remaining 90 unique web pages were categorized into academic, physician, commercial, medical professional, and non-identified groups. Quality was assessed using the DISCERN instrument, Journal of the American Medical Association (JAMA) Benchmark Criteria, and HONcode certification, while readability was evaluated with the Flesch-Kincaid Grade Level (FKGL) and Flesch-Kincaid Reading Score (FKRS). The SHS Content Score (SHS-CS) was also developed for a comprehensive content-specific evaluation.

Results: Academic websites had the highest quality scores, with DISCERN (52.10 ± 6.85), JAMA (3.48 ± 0.50), and SHS-CS (27.85 ± 2.15), but demonstrated lower readability (FKGL: 11.76 ± 0.40, FKRS: 21.45 ± 7.12). Commercial and non-identified websites scored lowest across all quality measures. Significant correlations were found between DISCERN and JAMA (r = 0.932, p = 0.000*), SHS-CS and DISCERN (r = 0.918, p = 0.000*), and a negative correlation with readability metrics (DISCERN vs. FKRS, r = -0.668, p = 0.000*).

Conclusion: The quality of SHS-related online information varies significantly across website types. While academic websites provide the highest quality content, they often lack readability. HONcode-certified websites exhibited superior quality but did not differ significantly in readability compared to non-certified sites. Future efforts should focus on improving the readability of high-quality health information.

## Introduction

The hallmark of snapping hip syndrome (SHS), which is additionally known as “coxa saltans,” is a noticeable or audible snapping sound originating from the hip joint [1]. The phenomenon may present as bilateral or unilateral and can be characterized as either painful or not painful, idiopathic, or posttraumatic. A “snapping” of the iliotibial band (ITB) or the iliopsoas tendon causes SHS. The internal kind of iliopsoas variant can be generated by externally rotating a flexed, abducted hip and then extending and internally rotating it [2]. The external iliotibial variation occurs when the ITB shifts across the greater trochanter from posterior to anterior when the hip transitions from extension to flexion [2]. The two pathologies, internal and external, are collectively referred to as “extra-articular.” Intra-articular pathologies, such as loose bodies, torn labrum, and fractures, can also lead to snapping hips, and these conditions are typically more detrimental than extra-articular issues [3].

Most instances do not cause any symptoms; thus, it is difficult to determine the frequency in the general population. However, it has been found to affect up to 10% of the population [1]. The incidence of painful snapping is elevated among those engaged in specific sports that need extensive hip range of motion, such as dancers in ballet, athletes, and soccer players [4]. Diagnosis is mostly clinical, backed by MRI or ultrasonic tests in case intra-articular disease is suspected [5].

In 2013, approximately 83.8% of households in the US possessed computers, while 74.4% engaged with the Internet [6]. Recently, over 90% of the US population are internet users, and around 77% own a smartphone [6]. There is still a wide range in the reliability and quality of health information available online, which can lead to the spread of false information and the use of insufficient self-management resources [7,8]. Verifying the reliability, comprehension, and quality of SHS-related online content is crucial for ensuring that patients get precise and useful instructions.

Using standardized measures such as the DISCERN instrument and the Journal of the American Medical Association (JAMA) standards, prior studies have evaluated online information quality for several orthopedic disorders, including rotator cuff tears, anterior cruciate ligament injuries, and scoliosis [9-11]. Though SHS is clinically relevant, to our knowledge, no studies have been published evaluating the efficacy of internet-based tools for SHS. By analyzing the contents, accuracy, and readability of SHS-related web content, the current study aimed to fill the knowledge gap.

## Materials and methods

**Figure 1:**
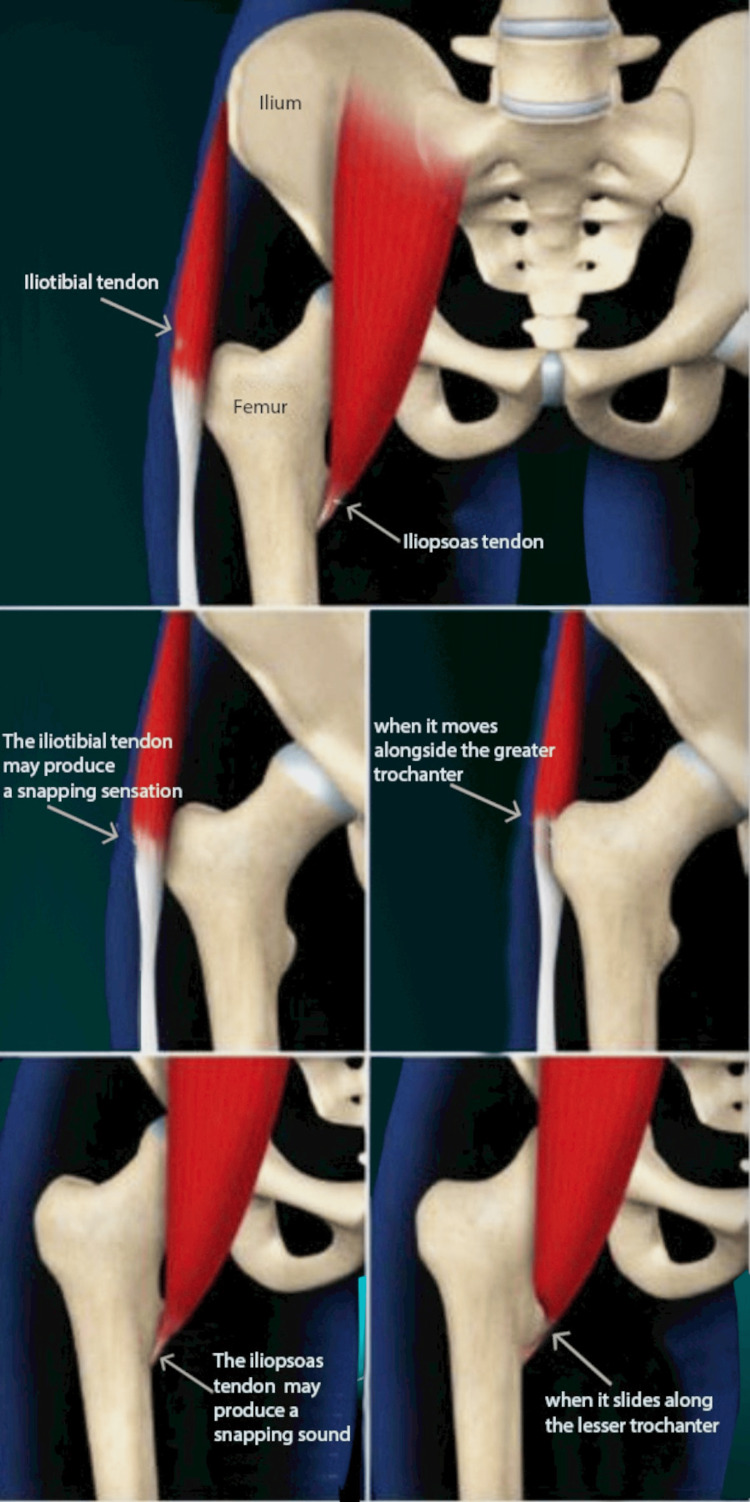
Pathophysiology of Snapping Hip Syndrome (SHS) The image illustrates the movement of the iliotibial band over the greater trochanter and the iliopsoas tendon over the lesser trochanter, which may lead to a snapping sensation. The image was created by the authors of this article.

Websites that are available to the public are the focus of this research. This entity does not engage with individuals or information about them, nor does it provide any reports related to them. Hence, informed consent and institutional review board authorization are unnecessary. Figure 1 illustrates the pathophysiology of SHS, demonstrating the movement of the ITB and iliopsoas tendon in relation to the greater and lesser trochanters.

Methodology for information retrieval

On January 24, 2025, the search engines Google, Bing, and Yahoo were employed to investigate the term “Snapping Hip Syndrome.” For analysis, the initial 30 URLs obtained from every search tool were chosen. The selected search engines accounted for over 96% of the entire market share in January 2025 [12]. The search was conducted in English and utilized the three most popular web engines. Payment walls and similar web pages, as well as duplicate ones, were excluded from consideration. Selection bias is an unavoidable challenge in web-based research due to constantly evolving search engine algorithms and ranking dynamics. To enhance transparency, all searches were performed at a predefined time using identical keywords. While fluctuations in search rankings may still influence results, no manual filtering or exclusion of websites was applied beyond the predefined selection criteria.

Classification of webpages

The authors placed websites into five categories: academic, physician, commercial, medical professional, and undefined. The academic sites were medical facilities and institutes qualified to claim academic affiliation or subject any item on their site for peer review. Physician and private practice sites covered websites run by individual doctors or small groups of experts. Commercial sites were ones with advertising or supporting certain designs. Designed for readers who are medical practitioners, medical professional sites were, generally speaking, instructive and informative in nature. Websites falling outside of any one of the predefined categories fell into the undefined category.

Assessment tools for quality and readability

Using three acknowledged criteria - the JAMA Benchmark Criteria (with scores ranging from 0 to 4), the DISCERN instrument (with scores ranging from 0 to 80), and certification from the Health on the Net Foundation (HONcode)-two competent hip surgeons decided on the quality [13-15]. An important and respected indicator of quality for health-related content found online is HONcode certification [14]. This initiative has established rigorous criteria for the generation and display of medical information in collaboration with the World Health Organization. These standards guarantee quality, transparency, and objectivity. This involved testing the certification and compliance of the HONcode label against previously listed websites. As they lay forth reliable standards for judging study quality, the evaluations presented in JAMA and DISCERN are crucial. This involves the evaluation of the aforementioned criteria used by JAMA, which mainly deals with the identification and clarity of authors, resources, disclosure, and date information [13]. Using 16 distinct criteria, DISCERN has assessed the reliability and validity of web-based resources [15]. Two independent doctors assessed and rated websites using criteria developed by JAMA and DISCERN. Looking at the data, a third author searched for any obvious changes in the score. We applied average values of JAMA and DISCERN for this study.

Subsequent to the selection of websites for further analysis, each was evaluated for readability. The assessment of readability involved calculating two distinct readability evaluations for each website through the online platform readable.com [16]. The Fleisch Reading Ease (FKRS) and the Fleisch Kincaid Grade (FKGL) were the scores that were calculated. The FKGL grade was established in the 1970s in order to assess the reading level necessary for understanding the text, which is determined by both sentence and word length [16]. FKRS is negatively connected with FKGL, where a higher score signifies more reading ease [16].

Lastly, to give a thorough and case-specific evaluation of internet information relevant to SHS, the authors of the research established the SHS Content Score (SHS-CS). This scoring system consists of 30 items grouped into four main groups: Diagnosis, Treatment, Patient Education, and Content Quality. Each item is scored as 1 (present) or 0 (absent), totaling 30 possible points (Table 1).

**Table 1:** Snapping Hip Syndrome Content Score (SHS-CS) Domains SHS: Snapping hip syndrome; IT: Iliotibial; MRI: Magnetic resonance imaging; FABER: Flexion, abduction, and external rotation

Domain	Score
Diagnosis (10 points)	
1. Definition of SHS	0/1
2. Differentiation between internal, external, and intra-articular snapping	0/1
3. Description of relevant anatomy (iliopsoas, IT band, etc.)	0/1
4. Common symptoms associated with SHS	0/1
5. Physical examination maneuvers (e.g., FABER test, Ober test)	0/1
6. Use of diagnostic imaging (e.g., MRI, ultrasound)	0/1
7. Differential diagnosis of hip snapping	0/1
8. Indications for specialist referral	0/1
9. Description of associated risk factors (e.g., overuse, anatomical variations)	0/1
10. Mention of prevalence and epidemiology	0/1
Treatment (10 points)	
11. Conservative management options (e.g., rest, activity modification)	0/1
12. Specific physical therapy exercises for SHS	0/1
13. Role of stretching and strengthening in management	0/1
14. Use of anti-inflammatory medications	0/1
15. Indications for corticosteroid injections	0/1
16. Description of surgical interventions (if applicable)	0/1
17. Postoperative rehabilitation protocols	0/1
18. Expected outcomes of different treatment approaches	0/1
19. Discussion of potential complications or recurrence	0/1
20. Evidence-based references supporting treatment recommendations	0/1
Patient Education (5 points)	
21. Clear explanation of the condition in lay terms	0/1
22. Use of visuals or diagrams to illustrate anatomy or exercises	0/1
23. Advice on lifestyle modifications to prevent recurrence	0/1
24. Accessibility features (e.g., simple language, multimedia content)	0/1
25. Provision of resources for further reading or support	0/1
Content Quality (5 points)	
26. Authorship and source credibility (e.g., physician-authored)	0/1
27. Disclosure of potential conflicts of interest	0/1
28. Currency of information (publication/update date)	0/1
29. Presence of references to peer-reviewed sources	0/1
30. Balanced, unbiased presentation of treatment options	0/1

## Results

**Figure 2:**
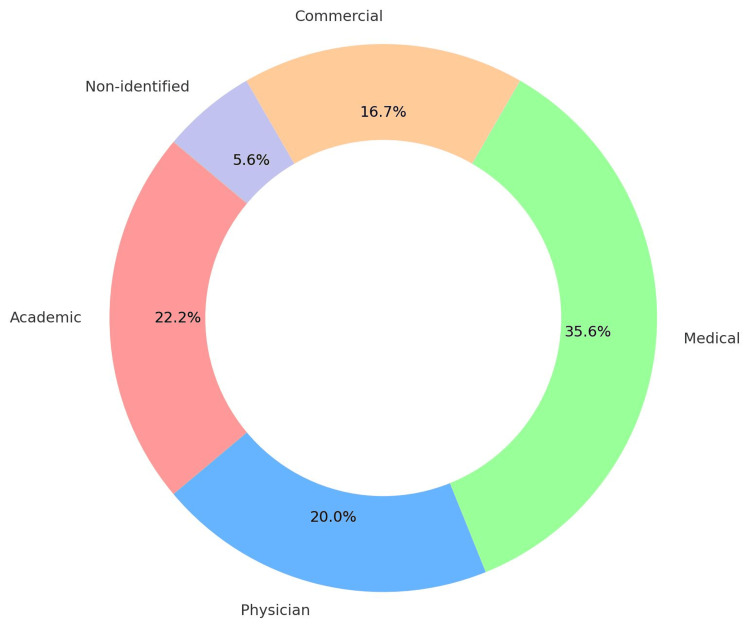
Website distribution based on sources The image was created by the authors of this article.

After deleting duplicates and irrelevant links, a total of 90 different web pages were taken into account in the final analysis. Figure 2 displays the distribution of websites.

Table 2 summarizes the results for each website in terms of DISCERN, JAMA, FKGL, FKRS, and SHS-CS.

**Table 2:** Minimum, Maximum, Mean, and Standard Deviation Values of the Assessment Tools JAMA: Journal of the American Medical Association; FKGL: Flesch-Kincaid Grade Level; FKRS: Flesch-Kincaid Reading Score; SHS-CS: Snapping Hip Syndrome Content Score

Assessment Tool	Min-Max	Mean ± SD
DISCERN Reviewer 1	19.2 – 62	36.15 ± 11.85
DISCERN Reviewer 2	18.4 – 63	37.42 ± 13.10
DISCERN Score	20.1 – 63	36.78 ± 12.02
JAMA Reviewer 1	1 – 4	2.05 ± 0.88
JAMA Reviewer 2	1 – 4	2.18 ± 1.09
JAMA Score	1 – 4	2.12 ± 0.98
FKGL	4.2 – 12.5	9.45 ± 2.15
FKRS	7.1 – 78.4	45.38 ± 18.90
SHS-CS	6 – 29	20.12 ± 7.48

The findings of different academic tools were evaluated and compared within several categories of websites, as indicated in Table 3.

**Table 3:** Category-Based Scores for Snapping Hip Syndrome p^1^: Kruskal-Wallis Test *p < 0.05 JAMA: Journal of the American Medical Association; FKGL: Flesch-Kincaid Grade Level; FKRS: Flesch-Kincaid Reading Score; SHS-CS: Snapping Hip Syndrome Content Score

Category	DISCERN Score Mean ± SD	JAMA Score Mean ± SD	FKGL Mean ± SD	FKRS Mean ± SD	SHS-CS Mean ± SD
Academic	52.10 ± 6.85	3.48 ± 0.50	11.76 ± 0.40	21.45 ± 7.12	27.85 ± 2.15
Physician	39.15 ± 8.74	2.42 ± 0.78	9.25 ± 1.88	44.75 ± 17.64	22.14 ± 5.32
Medical	32.95 ± 9.82	1.74 ± 0.69	8.61 ± 2.12	53.22 ± 15.18	17.82 ± 6.95
Commercial	23.54 ± 7.12	1.13 ± 0.31	7.65 ± 1.65	59.88 ± 11.02	12.34 ± 5.43
Non-identified	25.12 ± 7.85	1.25 ± 0.39	7.89 ± 1.74	57.45 ± 12.15	13.78 ± 5.87
p1	0.000*	0.000*	0.000*	0.000*	0.000*

The highest DISCERN scores (52.10 ± 6.85), JAMA scores (3.48 ± 0.50), and SHS-CS (27.85 ± 2.15) were recorded on academic websites. This shows that these websites have better content quality and greater reliability as well as more detailed information. These websites usually provide thorough descriptions of SHS, precise diagnostic criteria, and appropriate evidence-based treatment modalities. The high comprehensibility scores (FKGL: 11.76 ± 0.40, FKRS: 21.45 ± 7.12) portray the information to be detailed; however, it indicates high complexity, and thus a higher ability to read is needed.

The high ratings on DISCERN (39.15 ± 8.74) and SHS-CS (22.14 ± 5.32) show the credible and trustworthy quality of physician-written online pages, despite their little less thoroughness than academic websites. All medical web pages for medical professionals obtained moderate ratings in the grading system. DISCERN and JAMA both indicated that the presence of clinical data would not be patient-friendly, with scores of 32.95 ± 9.82 and 1.74 ± 0.69, respectively.

On the other hand, commercial websites had the worst values in all of these scores, recorded as DISCERN 23.54 ± 7.12, JAMA 1 1.13 ± 0.31, and SHS-CS 12.34 ± 5.43. While promoting their goods and services, these websites usually failed to include thorough medical information. While unspecified websites did better than commercial sites in terms of overall score (DISCERN: 25.12 ± 7.85, SHS-CS: 13.78 ± 5.87), they were still not able to meet the requirements for delivering trustworthy and thorough material.

**Figure 3:**
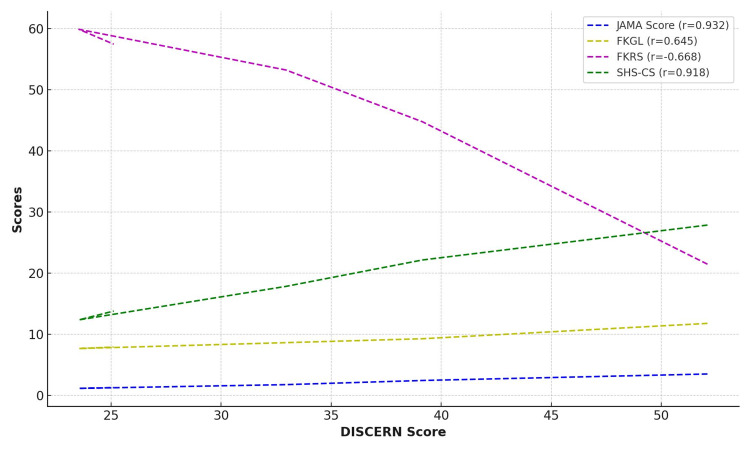
The correlation between DISCERN and other assessments JAMA: Journal of the American Medical Association; FKGL: Flesch-Kincaid Grade Level; FKRS: Flesch-Kincaid Reading Score; SHS-CS: Snapping Hip Syndrome Content Score The image was created by the authors of this article.

All three of the variables showed statistically significant correlations: DISCERN with JAMA scores (r = 0.932, p = 0.000*), SHS-CS with DISCERN (r = 0.918, p = 0.000*), and JAMA with SHS-CS (r = 0.874, p = 0.000*) (Table 4) (Figure 3).

**Table 4:** Correlation Analysis of Assessment Tools Spearman Rho Correlation Analysis *p < 0.05 JAMA: Journal of the American Medical Association; FKGL: Flesch-Kincaid Grade Level; FKRS: Flesch-Kincaid Reading Score; SHS-CS: Snapping Hip Syndrome Content Score; NA: Not Applicable

	DISCERN Score	JAMA Score	FKGL	FKRS	SHS-CS
DISCERN Score	NA	r = 0.932, p = 0.000*	r = 0.645, p = 0.000*	r = -0.668, p = 0.000*	r = 0.918, p = 0.000*
JAMA Score	NA	NA	r = 0.653, p = 0.000*	r = -0.675, p = 0.000*	r = 0.874, p = 0.000*
FKGL	NA	NA	NA	r = -0.949, p = 0.000*	r = 0.699, p = 0.000*
FKRS	NA	NA	NA	NA	r = -0.734, p = 0.000*
SHS-CS	NA	NA	NA	NA	NA

**Figure 4:**
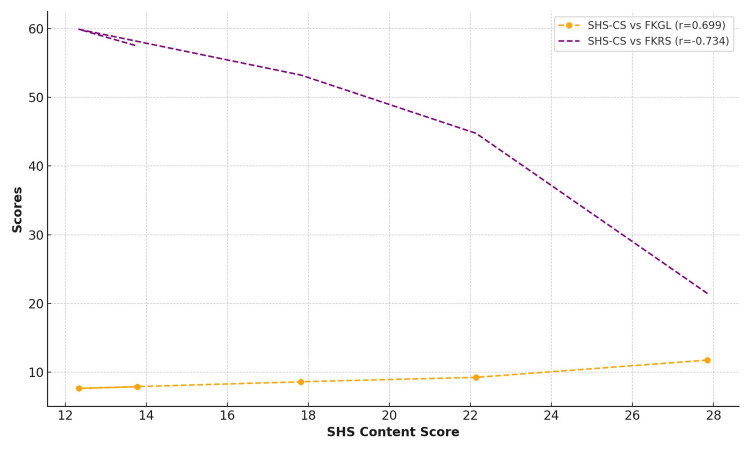
Correlation between SHS-CS scores, FKGL scores, and FKRS scores FKGL: Flesch-Kincaid Grade Level; FKRS: Flesch-Kincaid Reading Score; SHS-CS: Snapping Hip Syndrome Content Score The image was created by the authors of this article.

This suggests that across several evaluation methodologies, websites with greater quality consistently fared better. There was a negative correlation between quality ratings and readability scores as measured by FKGL and FKRS, suggesting that higher-quality content is typically more complex and problematic to read (Figure 4).

It is worth noting that extensive, high-quality medical information often neglects readability, as indicated by the positive correlation between FKGL and DISCERN (r = 0.645, p = 0.000*) and the negative correlation between FKRS and DISCERN (r = -0.668, p = 0.000*).

Table 5 ranks the scores in relation to HONcode certification presence.

**Table 5:** Assessment of Scores in Relation to the Existence of the HONcode Mann-Whitney U Test *p < 0.05 JAMA: Journal of the American Medical Association, FKGL: Flesch-Kincaid Grade Level, FKRS: Flesch-Kincaid Reading Score, SHS-CS: Snapping Hip Syndrome Content Score, HONcode: Health On the Net Code

Assessment Tool	AbsentMean ± SD	PresentMean ± SD	p-value
DISCERN Score	33.12 ± 12.15	45.78 ± 11.02	0.004*
JAMA Score	1.97 ± 1.02	2.73 ± 0.95	0.012*
FKGL	9.23 ± 2.31	9.15 ± 2.12	0.795
FKRS	46.19 ± 20.18	48.45 ± 17.33	0.732
SHS-CS	18.12 ± 7.85	24.01 ± 6.12	0.018*

Websites certified in HONcode showed much better DISCERN (45.78 ± 11.02 vs. 33.12 ± 12.15, p = 0.004*) and JAMA scores (2.73 ± 0.95 vs. 1.97 ± 1.02, p = 0.012*). This validates that sites authorized by HONcode offer more consistent and transparent health information. Reflecting a more comprehensive and ordered display of SHS-related material, SHS-CS was also much higher in HONcode-certified websites (24.01 ± 6.12 vs. 18.12 ± 7.85, p = 0.018*).

There are no significant differences in reading levels between students with HONcode certification and those without, as indicated by the lack of changes in FKGL (9.15 ± 2.12 vs. 9.23 ± 2.31, p = 0.795) and FKRS (48.45 ± 17.33 vs. 46.19 ± 20.8, p = 0.732). It appears that HONcode accreditation does not necessarily lead to increased reading convenience, even while it ensures higher reliability and quality of information. Online health information continues to face the issue of combining readability with content quality, as highlighted by these studies.

**Figure 5:**
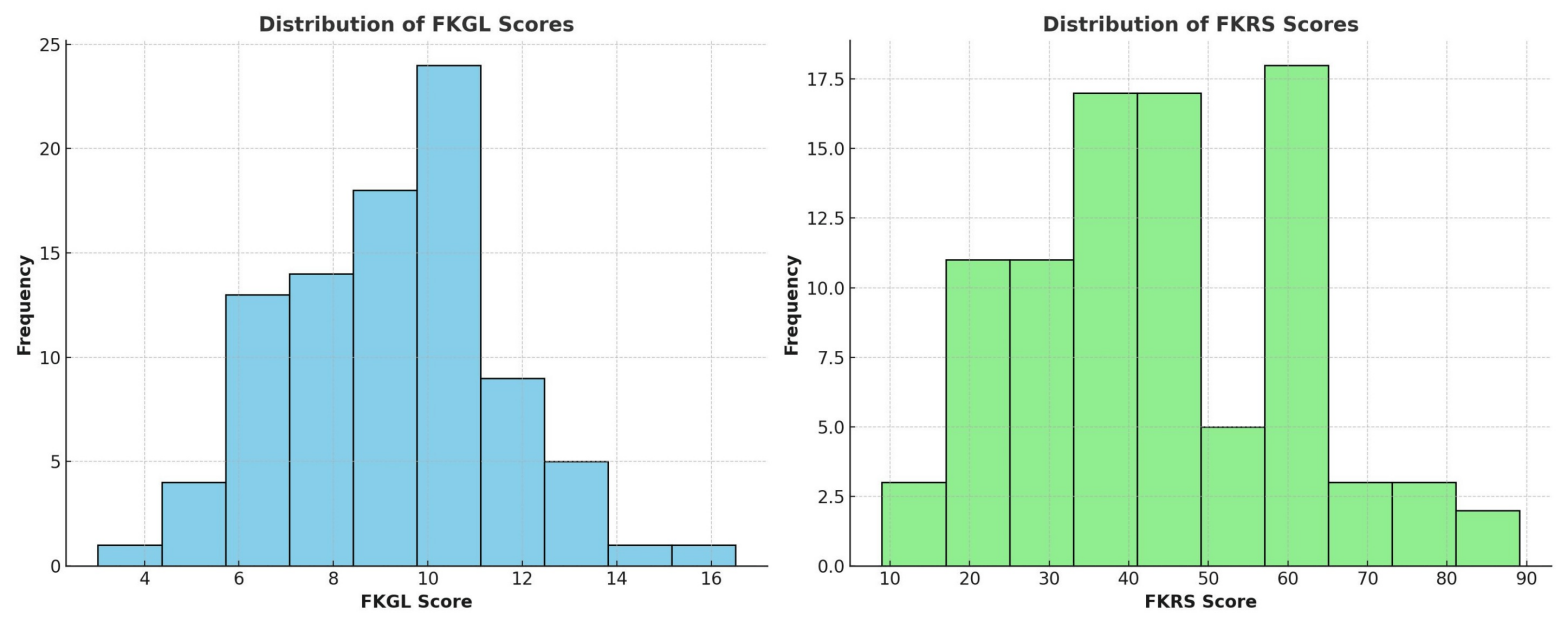
The distribution of readability scores across the evaluated websites FKGL: Flesch-Kincaid Grade Level; FKRS: Flesch-Kincaid Reading Score The image was created by the authors of this article.

The average FKGL score was 9.45 ± 2.15, and the average FKRS score was 45.38 ± 18.90, according to the current study (Figure 5). According to these results, the FKGL score is around 3.5 points greater than the American Medical Association (AMA) and National Institutes of Health (NIH)-recommended sixth-grade reading level.

## Discussion

The current study is the first of its kind to evaluate the reliability of SHS-related web resources. Academic websites were found to have the most reliable and accurate content, as shown by their high DISCERN, JAMA, and SHS-CS. Commercial and unspecified websites, in contrast, scored the lowest, indicating that the material on these sites was of poor quality. Strong linkages between the evaluation tools confirm their reliability; the negative correlation with readability highlights the challenge of delivering conveniently available high-quality information. Emphasizing the requirement of balancing accuracy with user-friendliness, HONcode-certified websites demonstrated greater quality but equivalent readability than non-certified sites.

The Internet has become an important provider of health data for patients, with both the total number of users and the proportion seeking medical data on the Internet rising over the past decade [17]. The proliferation of information availability is crucial for those wishing to make educated health decisions; nevertheless, the uncontrolled character of the Internet creates issues over the quality and material of online information. A systematic review indicated that data quality was a concern in 70% of studies evaluating the quality of health information available online [18].

Our research shows that there is a wide range in the quality of SHS-related material found online. All of the examined websites had a “poor” mean DISCERN score [19]. We discovered that several questions regularly had low scores when we looked at the DISCERN question-specific replies. Initially, websites frequently failed to delineate the purpose of the online material or the target population. Secondly, most websites failed to credit sources or provide independent reference resources for patients seeking further information. Finally, websites frequently failed to provide the publishing date or the date of the most recent material change.

Consistent with previous research [20,21], the present study revealed that the DISCERN, JAMA, FKGL, and SHS-CS scores in the academic group were considerably higher than those in other groups. According to McCormick et al. [20], the most often used sources of femoroacetabular impingement (FAI)-related content online were medical professional and commercial websites. These websites’ academic rigors and accessibility vary substantially. Like our findings, their research underlined that although commercial sites were often lacking in both quality and information, academic sources supplied the most dependable and thorough material. Raja et al. [22] investigated the quality and readability of online information on first metatarsophalangeal (MTP) joint fusion and found similar problems, with almost half of the assessed websites providing “poor” information. The mean DISCERN score in their study was 49.13 ± 5.76, which is comparable to the mean DISCERN score of our study (36.78 ± 12.02). Although academic websites had the highest DISCERN scores, they were difficult to understand for patients, reflecting our findings in online resources on SHS. Nassiri et al. [23] evaluated online information on Legg-Calvé-Perthes disease and reported generally low-quality information. In their study, mean DISCERN scores were reported to be comparable to those observed in our SHS study. While academic resources did provide the most reliable material on hip arthroscopy, Ellsworth et al. [17] discovered that the quality of internet resources was uneven overall. Our research on SHS confirms this, showing that academic websites are the most common sources of high-quality material.

Consistent with other research study [24], the average JAMA benchmark score was 2.12±0.98 on a roll of 4. A possible explanation for the poor JAMA results is that the majority of websites did not include any citations or helpful resources. We found a favorable relationship (p=0.000; p<0.05) between the DISCERN scores and the JAMA benchmark criteria. This could be due to the fact that the two items that make up the DISCERN score are associated with two crucial components of the JAMA benchmark criterion score: the availability of references and the date of publication.

The average FKGL score was 9.45 ± 2.15, and the average FKRS score was 45.38 ± 18.90, according to the current study. According to these results, the FKGL score is around 3.5 points greater than the AMA and NIH-recommended sixth-grade reading level. The study’s most notable finding is the negative link between readability and material quality, which poses a major obstacle. Complex vocabulary and thorough explanations are commonplace in high-quality medical information, making it challenging for the typical patient to comprehend. This is not an exclusive problem with SHS data; researchers have regularly found it in studies involving a wide range of orthopedic disorders [25-27]. According to a study on hip OA conducted by Lim et al. [26], the majority of websites were created at a level that was beyond what the average patient could understand. Their studies show that just a small fraction of websites meet the required sixth-grade reading level, implying that even if information may be correct, the general public cannot easily access it. Our study likewise revealed this trend: lower reading scores of higher-quality academic websites indicated a continuous challenge in balancing comprehensive medical information with layperson accessibility. According to a study conducted on the topic of hand osteoarthritis by Hong et al. [27], the majority of online resources are poorly written and difficult for the ordinary person to understand. Their results reflect our opinions, particularly in relation to the use of more technical language by academic and physician-authored web pages, which reduces patient involvement and understanding. It is crucial to work on making the material more readable without sacrificing its accuracy. Research has shown that patients can better understand medical information when physicians use visual aids, minimize medical jargon, and use plain, simple language. Websites including graphics and sequential descriptions of methods are often more engaging and comprehensible, as noted in the literature [6,20].

Online articles in this study with a HONcode had a better quality, which is consistent with previous research [28,29]. This lends credence to the assumption that websites bearing this designation give reliable, high-quality information. According to this research, just 24.6% of websites had a HON code, while 76.4% did not. In line with other studies, HONcode certification has been shown to be an accurate measure of content quality. According to Ellsworth et al. [17], websites that are certified by HONcode tend to get higher scores in DISCERN assessments, even if they may not have better readability. These results are supported by our study, which found that websites that were certified by HONcode were more forthcoming and truthful with their content, but they were not noticeably easier to read than non-certified sites.

Limitations and strengths

The current study has some drawbacks. First, the focus of this study was on the English language websites, which cannot represent the quality and readability of SHS-related material in other languages and hence may not allow the generalizability of these results. Second, since the internet is a dynamic entity, the content on the assessed websites can change at any time, and this will affect the repeatability of results. Third, though the study applied standardized methods of evaluation such as DISCERN and JAMA, subjective bias cannot be avoided in giving a score. Further, not evaluated in this study were the recently increased use of health knowledge through multimedia materials, such as interactive modules or movies. Lastly, a key limitation of this study is the influence of search engine algorithms on website rankings. Factors like user engagement, location, and personalization can cause variability in search results. While our standardized search strategy aimed to minimize this, it remains an inherent limitation in web-based research.

Despite such limitations, the strengths of our study remain salient. The current study is the first-ever comprehensive assessment of online information on SHS using strong and validated assessment tools such as DISCERN, JAMA benchmarks, and SHS-CS. The inclusion of multiple types of websites (academic, physician, commercial, and unspecified) provides a broad overview of the current online landscape. Moreover, the correlation analysis between different quality assessment tools adds to the methodological rigor, offering insights into the consistency and reliability of these measures.

## Conclusions

This is the first comprehensive study conducted in the assessment of the quality and readability of online resources related to SHS. Our research shows that there is a lot of variation between different kinds of websites; for example, academic websites tend to have the best content but poor readability. The content quality of commercial and unspecified websites was poorer, which raises questions about the dependability of health information that is freely accessible online.

While HONcode certification was associated with better quality, it did not consistently improve readability. The dependability of these evaluation methods is highlighted by the substantial connection between DISCERN, JAMA, and SHS-CS. However, there is still a recurring issue in finding an accessible balance between accuracy and readability. To enhance the readability of online medical content, the use of visual aids, minimizing medical jargon, and adopting plain, simple language can be beneficial. Websites that incorporate graphics and provide step-by-step explanations improve user engagement and comprehension, making complex medical information more accessible to a wider audience. Improving the readability of high-quality materials to better assist informed patient decision-making should be the focus of future initiatives.
